# Integrated strategic planning and multi-criteria decision-making framework with its application to agricultural water management

**DOI:** 10.1038/s41598-022-12194-5

**Published:** 2022-05-19

**Authors:** Ahmad Radmehr, Omid Bozorg-Haddad, Hugo A. Loáiciga

**Affiliations:** 1 Department of Irrigation and Reclamation Engineering, Faculty of Agricultural Engineering and Technology, College of Agriculture and Natural Resources, University of Tehran, Karaj, 3158777871 Tehran Iran; 2 Department of Geography, University of California, Santa Barbara, CA 93106 USA

**Keywords:** Climate sciences, Ecology, Environmental sciences, Environmental social sciences, Hydrology, Natural hazards, Engineering, Mathematics and computing

## Abstract

Sustainable water resources management involves social, economic, environmental, water use, and resources factors. This study proposes a new framework of strategic planning with multi-criteria decision-making to develop sustainable water management alternatives for large scale water resources systems. A fuzzy multi-criteria decision-making model is developed to rank regional management alternatives for agricultural water management considering water-resources sustainability criteria. The decision-making model combines hierarchical analysis and the fuzzy Technique for Order of Preference by Similarity to Ideal Solution (TOPSIS). The management alternatives were presented spatially in the form of zoning maps at the level of irrigation zones of the study area. The results show that the irrigation management zone No.3 (alternative A3) was ranked first based on agricultural water demand and supply management in five among seven available scenarios, in which the scenarios represents a possible combination of weights assigned to the weighing criteria. Specifically, the results show that irrigation management zone No.3 (alternative A3) achieved the best ranking values of 0.151, 0.169, 0.152, 0.174 and 0.164 with respect to scenarios 1, 4, 5, 6 and 7, respectively. However, irrigation management zone No.2 (alternative A2) achieved the best values of 0.152 and 0.150 with respect to the second and third scenarios, respectively. The model results identify the best management alternatives for agricultural water management in large-scale irrigation and drainage networks.

## Introduction

Water management is becoming more challenging by the effects of climate change, population growth, and severe competition for water by the municipal, agricultural, industrial, and energy sectors^3,13,20,24,57^. Accordingly, integrated water resources management focuses on water demand and supply management to achieve sustainable development. Water is a scarce resource essential for societal survival and functioning. This makes the application of integrated water resources management essential to cope with scarcity and the challenges posed by climate change and increased water demand to by expanding economies^26^. A conceptual framework combining integrated landscape management (ILM) and institutional design principles (IDP) perspectives was applied to analyze cooperation initiatives involving water suppliers and agricultural stakeholders from agricultural wastewater^5^. A national drought risk assessment for agricultural lands taking into account the complex interaction between different risk components was presented^40^. The research showed that crop diversification, crop pattern management, and conjunctive (i.e., surface water and groundwater) water management can be effective in improving agricultural water^18,48^.

The management of today’s complex water supply and demand systems rely on assessment models combining climatic, social, economic, and environmental factors. A model was developed using the concept of risk by identifying hazards, exposure, and vulnerability^34^. The vulnerability was classified into two domains, i.e., sensitivity and adaptive capacity, and two spheres, natural/built environment and human environment. A geographical information system modeling and satellite data were developed for water management in agricultural areas by modulating the irrigation water demand based on several vegetation indices^2^. The water allocation rules were evaluated among water user groups considering environmental, economic, and social criteria involving agricultural water user groups across France^51^. Transferring of irrigation management was defined as the complete or partial transfer of responsibility for management and investment in irrigation systems from government institutions to water users and non-governmental organizations (NGOs)^66^. A combination of the Adaptation Pathways approach was used with the Soil and Water Assessment Tool (SWAT) to assess the actions under different climate conditions^6^.

Conjunctive management requires a strong institutional capacity, which can be achieved through regional planning, based on a sound understanding of the interactions between surface water and groundwater^65^. Sustainability in basins with existing irrigation and drainage networks requires a strategic planning according to sustainable development principles^38^. Strategic planning refers to an organizational infrastructure that prioritizes plans and maximizes potential opportunities and benefits^19^. Sustainable development achieves present economic, environmental and social needs while fulfilling the needs of future generations. The lack of strategic vision with respect to sustainability practices and goals was discussed^9^. A SWOT analysis consists of well-structured strategic planning to assess the status of a system by evaluating its strengths (S), weaknesses (W), opportunities (O), and threats (T)^58^. A review of works based on SWOT analysis was reported^25^. A strategic approach was applied to water management in Africa with SWOT^22^. Strategic planning approaches were analyzed in Austrian flood-risk management by identifying background conditions to facilitate scaling and replication of catchment regional planning tools in flood-prone areas^60^. A raster-based regional conservation action planning tool was developed for prioritizing local and regional scale conservation actions in heterogeneous landscapes^61^.

A stochastic method was developed to determine the water availability in agricultural lands that resulted from drought management plans^47^. A regional optimization model of crop water consumption using cellular automation (CA), crop suitability (CS), and a regional distributed crop water use model was applied to improve irrigation benefits in the context of regional water management^28^. A study was reported to determine deficiencies in irrigation networks and remediation measures^1^.

Multi-criteria decision making (MCDM) is a branch of operations research that provides methods for choosing among alternatives ranked by multiple criteria. The Analytic Hierarchy Process (AHP) is a widely used decision-making tool in various multi-criteria decision-making problems^15^. The AHP, is an approach that uses ratio comparisons among attributes and alternatives^54^. A method of scaling ratios using the principal eigenvector of a positive pairwise comparison matrix was proposed^53^. This work defines and measures the consistency of the pairwise comparison matrix by an expression involving the average of the non-principal eigenvalues.

The literature on methods and applications of Multiple Attribute Decision Making (MADM) has been reviewed and classified systematically^30^. A review of the TOPSIS method for decision making was presented^70^. A new step‐wise weight assessment ratio analysis was introduced to determine the criteria weights in decision making problems^33^. The weights of the criteria were calculated using the integrated Stepwise Weight Assessment Ratio Analysis (SWARA)-SWARA-TODIM (an acronym in Portuguese for Interactive Multi-Criteria Decision Making) multi-criteria decision-making (MCDM) method^52^. The weighting methods in decision making process including the DEMATEL (Decision Making Trial and Evaluation Laboratory) and BWM (best worst method) was applied to achieve the importance of supplier criteria in a combined manner^67^. The fuzzy set in the form of a class of objects was introduced with a continuum of grades of membership^69^. The fuzzy extension of the AHP method was introduced^64^. Fuzzy TOPSIS method was applied for decision-making process^17^. The model integrating SWARA and Additive Ratio Assessment (ARAS) methods was introduced under uncertainty^32^. A new decision-making approach was developed by measuring attractiveness through a categorical-based evaluation technique and a new combinative distance-based evaluation method in a supplier selection problem during the COVID-19 pandemic^44^. The Level-based weight assessment (LBWA) in fuzzy environment was developed using actual score measures of the picture fuzzy numbers^11^. A novel extension of a developed multi criteria decision making (MCDM) algorithm known as the preference ranking on the basis of ideal-average distance method in fuzzy environment was applied to address a real-life complex decision making problem in social science research^12^. A comparative analysis of supply chain performances of leading healthcare organizations in India with three MCDM frameworks was reported^10^.

Uncertainty analysis was conducted using an integrated fuzzy lambda–tau and fuzzy multi criteria decision-making method^45^. The integrated Fermatean fuzzy information-based decision-making method was introduced based on the removal effects of criteria and the additive ratio assessment methods, and applied it to a food waste treatment technology selection problem^49^. A triangular intuitionistic fuzzy linear programming model was proposed for planning of sustainable production system in Baluchistan, Pakistan^35^. A fuzzy multi-criteria group decision-making model was investigated for watershed ecological risk management^21^. A fuzzy-TOPSIS-world open account (OWA)-based model was developed to identify the impacts of parameters influencing the water quality failure (WQF) potential^31^. A scenario-based fuzzy interval programming approach was developed for planning agricultural water, energy, food, and crop area management^71^. Game theory was applied for solving decision making problems. The method was applied to construction site selection, and demonstrated that game theory can be applied for supporting decision in a competitive environment^46^. SWOT analysis can be improved by combining it with MCDM^37^. The Analytic Hierarchy Process (AHP) and the Analytical Network Process (ANP) analysis have been combined with SWOT analysis^16,59,68^. Multiple criteria group decision making applied for prioritizing SWOT factors^23^.

Despite numerous studies on sustainable water management by researchers^27,36,42,43,50,62,63^ and research on sustainability principles^4^, sustainable agricultural water management at the local level and scale has received less attention. Studies by the Organization for Economic Co-operation and Development (OECD) on water sustainability indicators show that analysis at the local level and scale is necessary to demonstrate the effectiveness of the principles of water sustainability^41^.

The analysis of large-scale water resource systems involving multiple components, resources, stakeholders, reservoirs, small irrigation reservoirs, and water transfer schemes is a complex process. This work develops and applies a conceptual framework for sustainable agricultural water use and supply by applying regional management alternatives at multiple spatial scales. The framework is applied to a large scale water resources system considering social, economic and environmental factors. The framework applies conceptual and analytical methods to sustainable agricultural water management relying on strategic planning and regional multi-criteria decision-making. Previous works have evaluated the sustainability of water resources from different perspectives and methods. This study is novel in its introduction of a framework that measures the sustainability of large-scale agricultural water systems relying on regional management plans.

## Method

**Figure 1:**
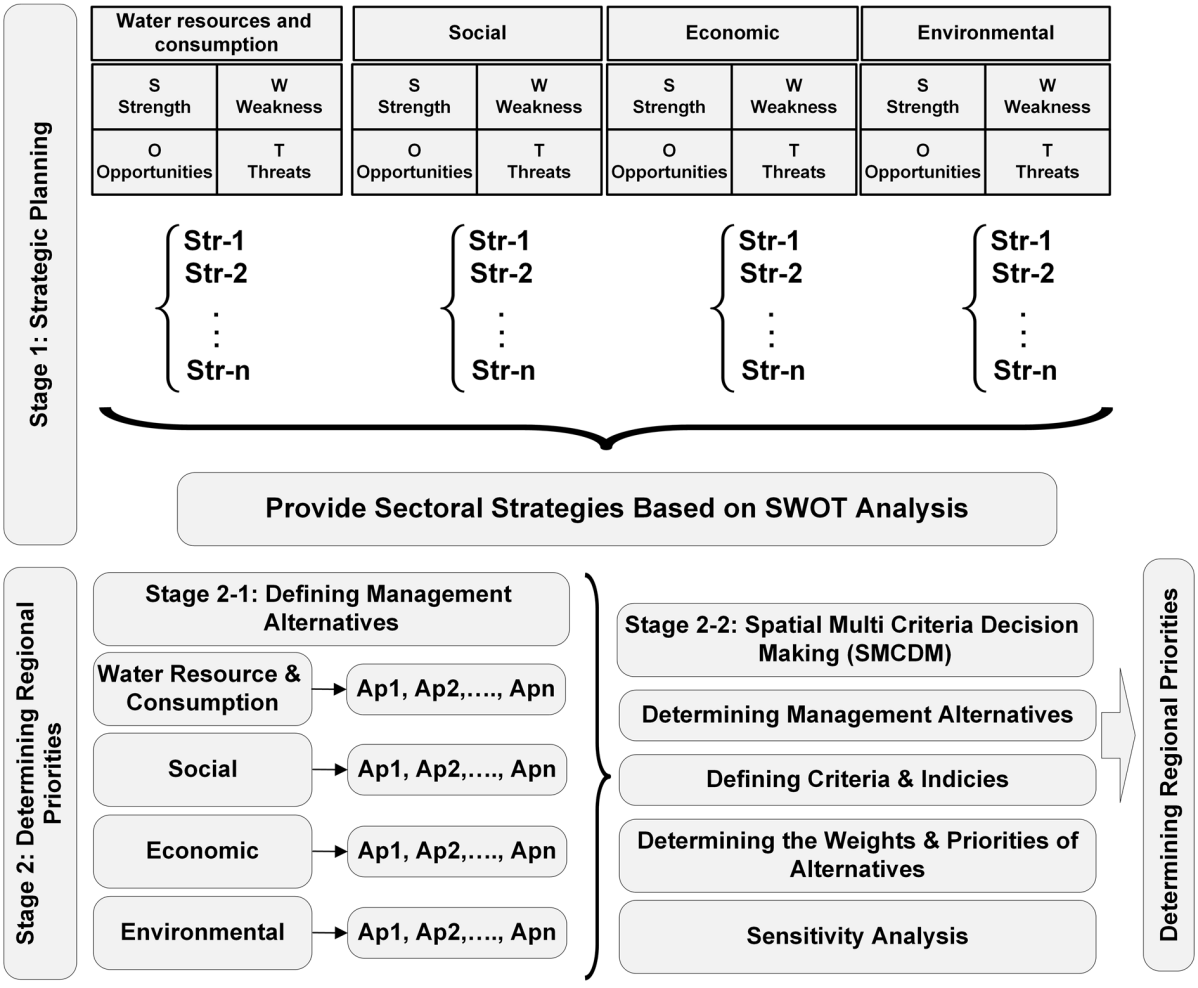
The integrated framework of strategic planning and spatial multi-criteria decision-making.

This section presents the conceptual framework for agricultural water demand and supply management and explains how to apply the conceptual framework for developing regional management alternatives (see Fig. 1). It is seen in Fig. 1 that the conceptual framework consists of two steps, namely, strategic planning and determining the regional priorities, which are explained below. Previous studies have established that entrenched challenges to water resources planning and management are common^8^. Effective implementation of integrated water policies is not common, and has led to a policy implementation gap that leads to incapacity in translating policy into action^7^. This work contributes to closing that gap.

### Strategic planning

The main purpose of strategic planning is to identify and analyze internal factors (strengths and weaknesses), external factors (opportunities and threats), and to formulate management alternatives for sustainable development of agricultural water management. The strategic planning stage considers agricultural water use and sources, social, environmental, and economic issues. The evaluation of the internal and external factors and determining the strengths, weaknesses, opportunities, and threats, and the current status of water resources leads to the formulation of sustainable agricultural water management plans, which is the basis for determining the regional priorities in the form of regional management alternatives.

### Determining regional priorities based on multi-criteria decision models

Spatial multi-criteria decision making analysis integrates spatial and non-spatial data and incorporates them in the decision-making process. This is accomplished by defining the relationship between input and output maps where by the spatial data and the priorities of the decision makers are accounted for and analyzed according to the rules of decision making^39^. The selected management alternatives defined in the first stage are formulated as a set of regional management alternatives for sustainable development of agricultural water management. Notice therefore that the output of the first stage is a set of management alternatives for sustainable development of agricultural water management, which constitutes the basis for defining regional management alternatives in the second stage. The management alternatives can be structural, non-structural, or a combination of both, which are addressed in terms of water demand and supply management.

#### Multi-criteria analysis of regional management alternatives for agricultural water demand and supply

This work implements multi-criteria decision making models to prioritize the irrigation management zones in terms of regional management alternatives for agricultural water demand and supply management. The proposed model implemented to prioritize the irrigation management zones is a combination of hierarchical analysis and the TOPSIS in a fuzzy environment.

## Study area

**Figure 2:**
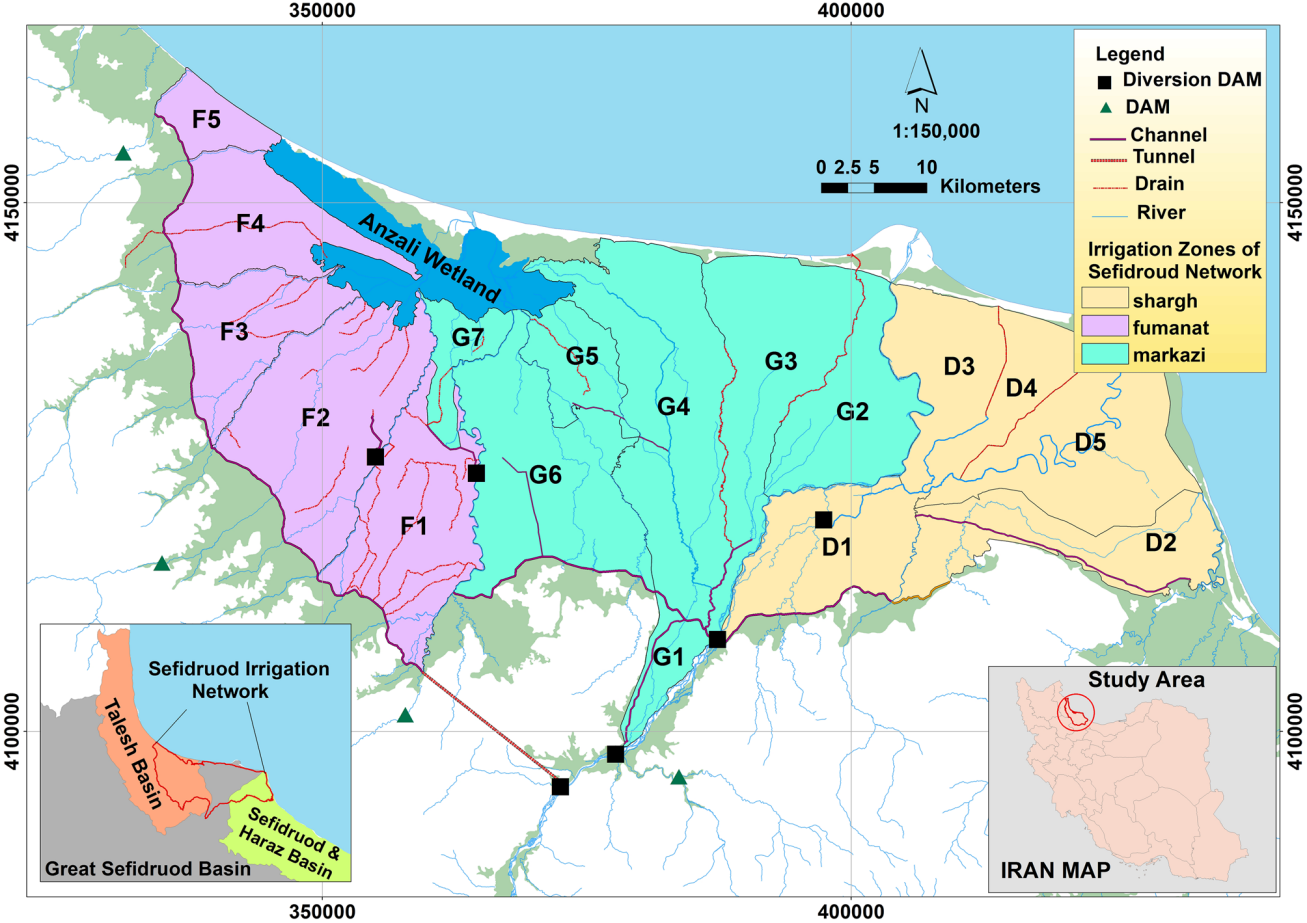
The location of the study area. (Figure created in ArcGIS 10.4 ESRI, http://www.esri.com).

The study area is the *Sefidroud* irrigation and drainage network, Iran, with an area of 284,000 hectares (Fig. 2). The irrigation network is divided into three irrigation management zones, namely, the *Markazi, Fumanat*, and *Shargh* irrigation zones, which are divided into 17 irrigation units, 10 of which have modern irrigation and 7 have traditional irrigation system. There are about 300,000 water users in the *Sefidroud* irrigation and drainage network and the main crop of the irrigation network is rice. About 94% of the total cultivated agricultural land is dedicated to rice fields. The main source of water supply for the *Sefidroud* irrigation and drainage network is *Sefidroud* Dam. There are other sources of water for the *Sefidroud* irrigation and drainage network, such as local rivers, farm wastewater, small irrigation reservoirs, and groundwater.

The *Sefidroud* irrigation network covers parts of three rivers basins in Iran, and is located in the downstream area of *Sefidroud* river basin. The *Sefidroud* basin covers eight provinces of Iran where there are regional conflicts concerning the management of the *Sefidroud* irrigation network.

## Results and application of the approach

This section describes the following topics:

- Analysis and evaluation of agricultural water use and resources in the study area.
- Study and analysis of internal factors (strengths and weaknesses) and external factors (opportunities and threats) related to agricultural water management in the study area.
- Determining the regional management alternatives of agricultural water demand and supply management.
- Multi-criteria analysis of the regional management alternatives of agricultural water demand and supply management.

### Analysis and evaluation of agricultural water use

**Figure 3:**
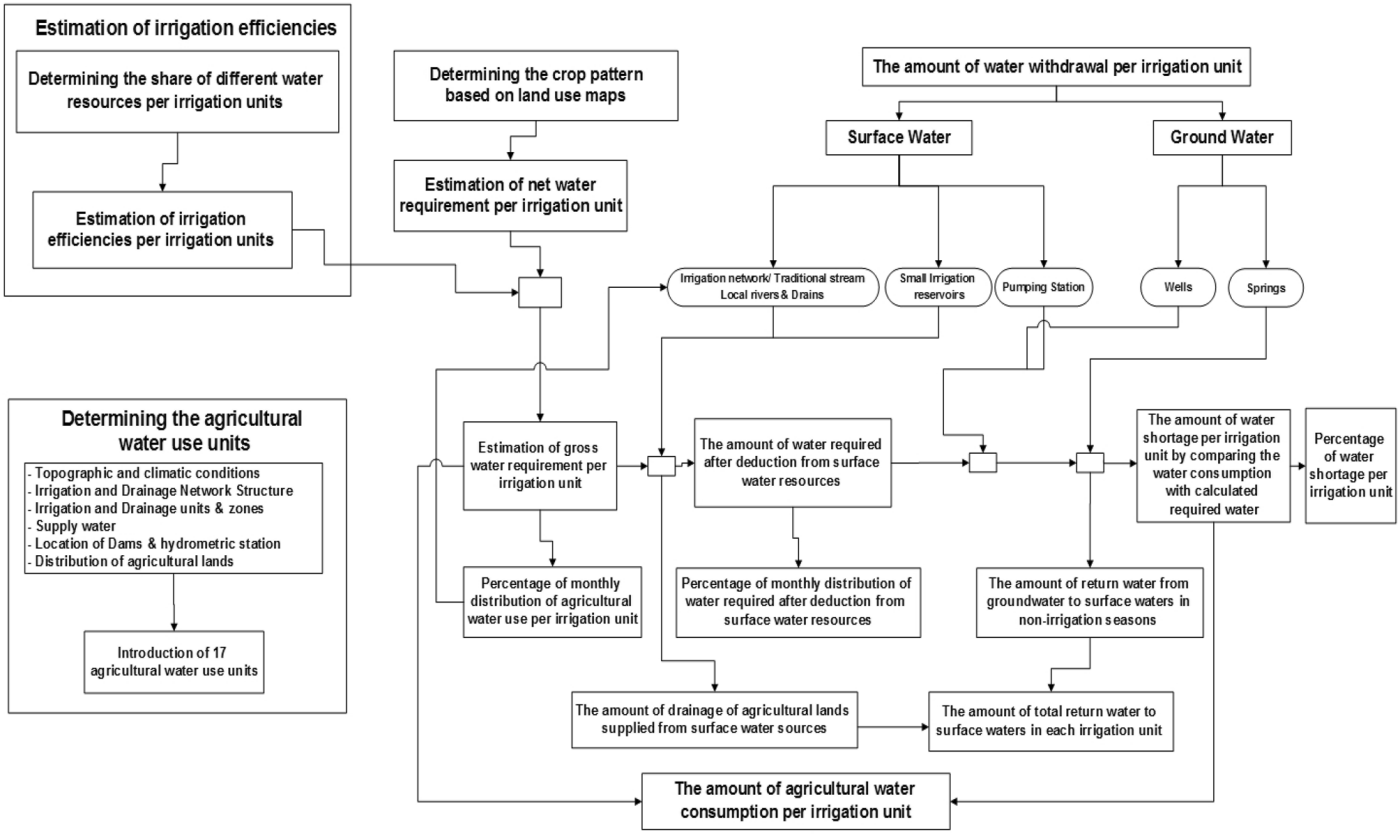
The method of agricultural water-use analysis.

The type of available water resources (*Sefidroud* network, local rivers, drainage, small irrigation reservoirs and groundwater resources), the crop pattern and quality of soil and water sources vary throughout the study area. Therefore, a database of water-use statistics was prepared to estimate the water use by agricultural lands within the *Sefidroud* irrigation and drainage network. The water use in the agricultural lands is a function of various factors such as the type of water resources, the method of water conveyance and distribution, the irrigation method, the type of crop products, climatic conditions, soil type, management practice, and others. Therefore, estimating the amount of water use in the agricultural areas in the study area is beset by complexity (Fig. 3).

The inputs to the agricultural water use model are (a) the cultivated area and crop pattern of irrigated lands, (b) the crop water requirements, (c) the irrigation efficiencies and (d) the surface and ground water withdrawal data. The agricultural water use analytical model calculates water use in each irrigation unit by comparing the water requirements of the crop pattern with the water withdrawals of surface water and groundwater. The outputs from this model are actual water use, the contributions of surface and groundwater to water use and the volumes of return flow.

**Figure 4:**
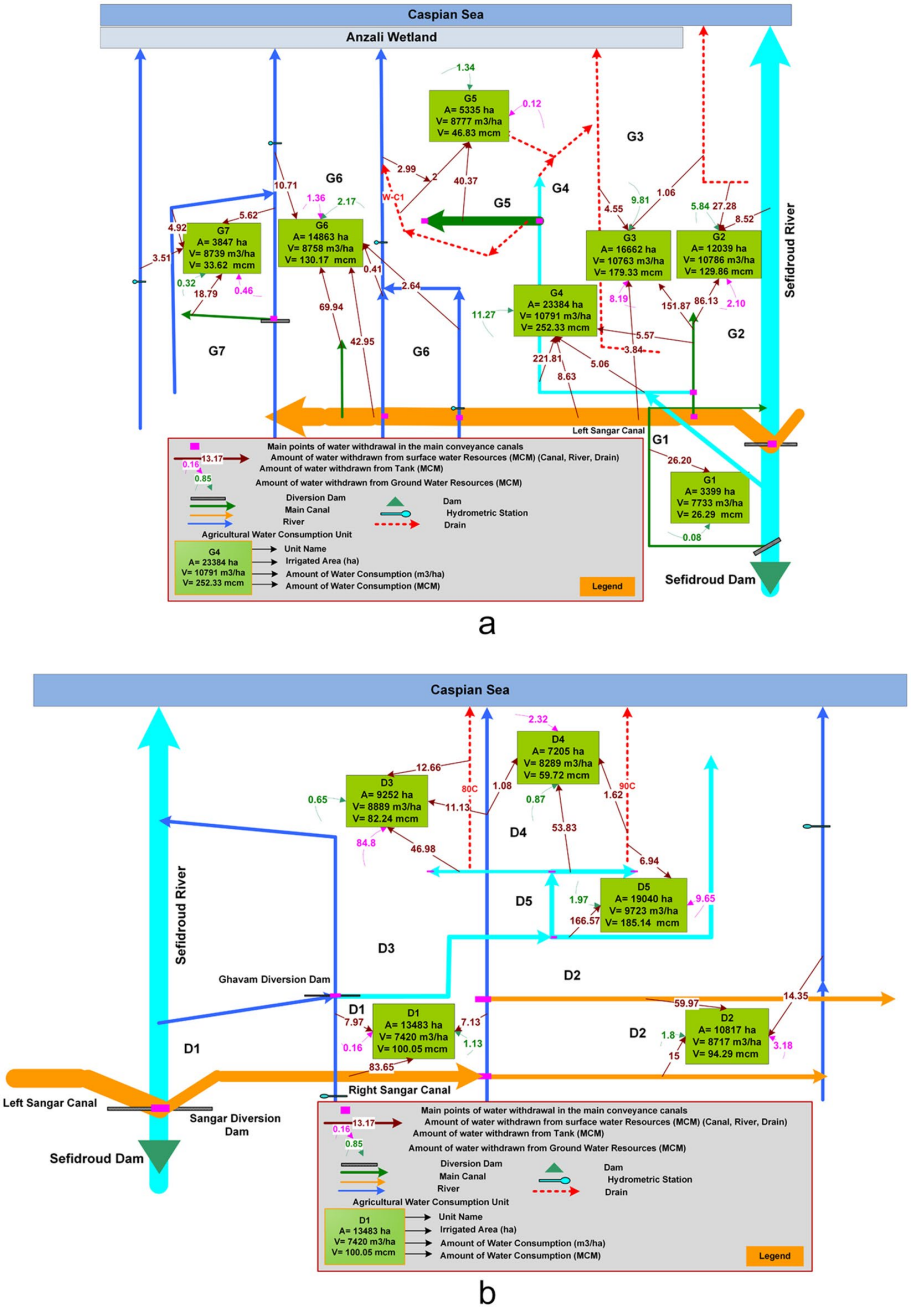

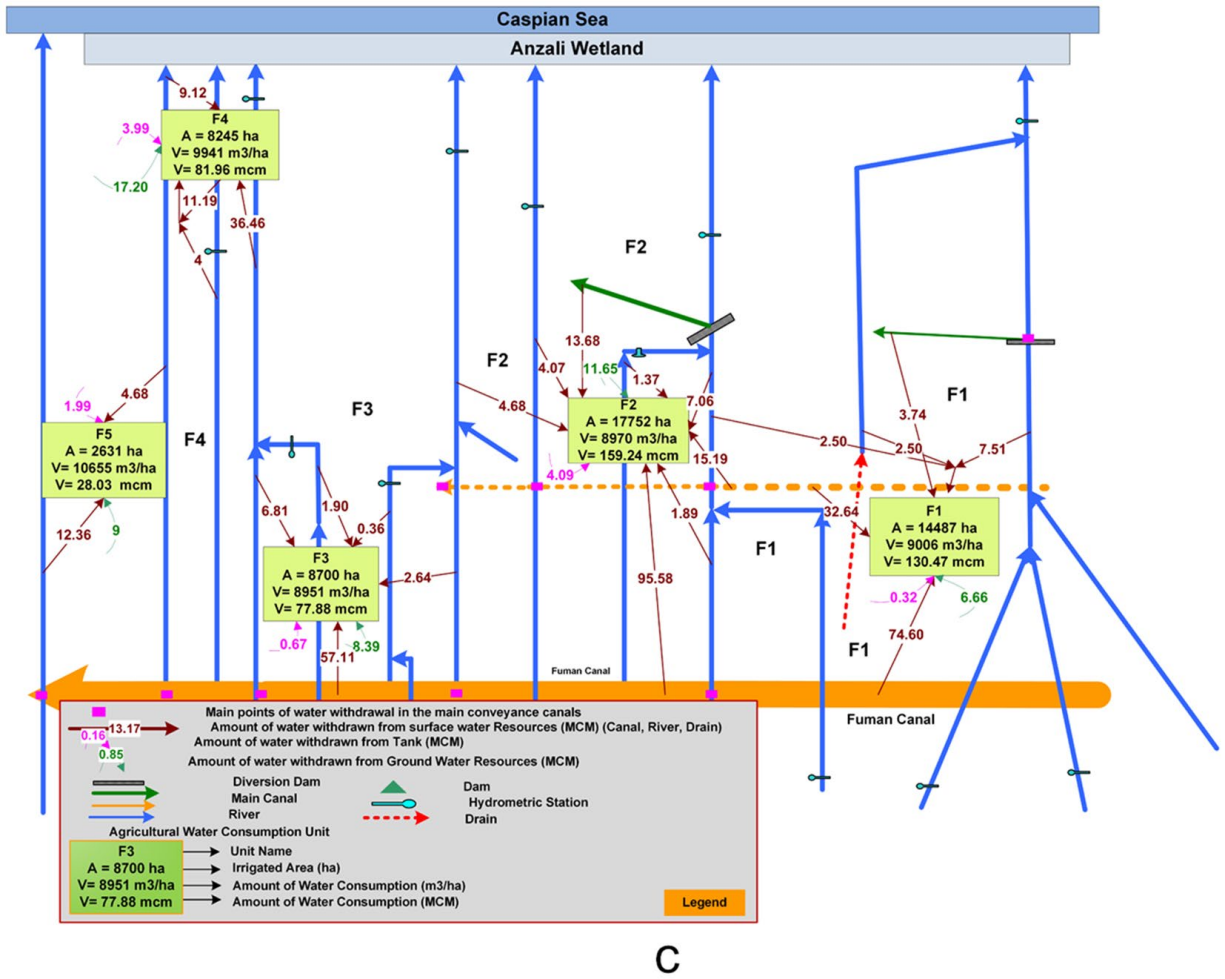
Detailed discription of agricultural water use from different water sources in the (**a**) *Markazi*, (**b**) *Shargh*, (**c**) *Fumanat* irrigation management zone.

The details of agricultural water use from different water sources (i.e., the *Sefidroud* dam and its related channels, local rivers, farm wastewater, small irrigation reservoirs, and groundwater) within the irrigated units of the *Sefidroud* irrigation network are depicted in Fig. 4 and listed in Table 1 for three irrigation management zones. It can be seen in Table 1 that the cultivated area of paddy fields in the *Sefidroud* irrigation and drainage network has been estimated at about 179,181 hectares. The total annual water use of cultivated area in *Sefidroud* irrigation and drainage network is about 1.8 billion cubic meters, of which about 1707 million cubic meters (95%) are surface water and 90 million cubic meters (5%) are groundwater. Of the total volume of surface water use about 1.4 billion cubic meters are from the *Sefidroud* dam and related canals, 260 million cubic meters from local rivers and farm wastewater, and about 47 million cubic meters from small irrigation reservoirs. The average volume of water use in the 191,141 hectares of irrigated lands of the *Sefidroud* irrigation and drainage network equals 9404 cubic meters per hectare.

**Table 1:** Agricultural water use in the irrigation management zones of the *Sefidroud* irrigation network.

No	*Sefidroud* irrigation zones	Irrigated area (ha)	Water supply resources	Water volume (10^6^ m^3^)
1	*Shargh* irrigation zone	59,797	*Sefidroud* irrigation network	426
			Local rivers	65
			Small reservoirs	24
			Total surface water use	515
			Groundwater use	6
			Total water use	521
2	*Fumanat* irrigation zone	51,815	*Sefidroud* irrigation network	293
			Local rivers	121
			Small reservoirs	11
			Total surface water use	425
			Groundwater use	53
			Total water use	478
3	*Markazi* irrigation zone	79,529	*Sefidroud* irrigation network	681
			Local rivers	74
			Small reservoirs	12
			Total surface water use	768
			Groundwater use	31
			Total water use	798
*Sefidroud* irrigation network		191,141	*Sefidroud* irrigation network	1400
			Local rivers	260
			Small reservoirs	47
			Total surface water use	1707
			Groundwater use	90
			Total water use	1797

### Analysis of internal and external factors pertinent to agricultural water management

SWOT analysis was introduced as a tool for complex water resources management (Thaler et al. 2020). This study separates internal and external factors by the geographical boundary of the irrigation network. Thus, the factors under the management of *Sefidroud* irrigation and drainage network are considered internal factors and the others are considered external factors. The main internal and external factors related to agricultural water management in the *Sefidroud* irrigation and drainage network are presented in Table 2.

**Table 2:** Main internal and external factors related to agricultural water management in *Sefidroud* irrigation and drainage network.

Internal factors (I.F)*
I.F-1: Strengthen the irrigation management institutions
I.F-2: Rehabilitation of small irrigation reservoirs with multi-purpose use
I.F-3: Conjunctive use of surface and groundwater resources
I.F-4: Intermittent irrigation method within paddy fields
I.F-5: Improper implementation of the irrigation development plans in seven irrigation units
I.F-6: Inappropriate operation and maintenance of irrigation and drainage network
I.F-7: Lack of necessary infrastructure for agricultural water delivery in the irrigation network
I.F-8: Failure to establish local participatory water management institutions
I.F-9: Lack of sufficient motivation among farmers to establish water user association (WUA)
I.F-10: The difficulty of pricing of agricultural water rights within the irrigation network
I.F-11: Lack of monitoring system of the agricultural water use
I.F-12: Lack of empowerment of the water users association (WUA)
I.F-13: Strengthen the irrigation management institutions
I.F-14: Rehabilitation of small irrigation reservoirs with multi-purpose use
I.F-15: Conjunctive use of surface and groundwater resources
I.F-16: Intermittent irrigation method within paddy fields
I.F-17: Improper implementation of the irrigation development plans in seven irrigation units
I.F-18: Inappropriate operation and maintenance of irrigation and drainage network
I.F-19: Lack of necessary infrastructure for agricultural water delivery in the irrigation network
I.F-20: Failure to establish local participatory water management institutions
I.F-21: Lack of sufficient motivation among farmers to establish water user association (WUA)
I.F-22: The difficulty of pricing of agricultural water rights within the irrigation network
External factors (E.F)**
E.F-1: Capacity of the internal water resources in the study area including local rivers and drains, small irrigation reservoirs and groundwater resources to supply agricultural water, especially under drought condition
E.F-2: Possibility of using agricultural return flow to supply irrigation water
E.F-3: Rules, procedures, standards, and technical guidelines for agricultural water resources management within the irrigation network
E.F-4: Upstream water resources development plans in the study area and its effects on reducing the water delivered to the irrigation network
E.F-5: Land use change and conversion of paddy fields into aquaculture ponds and increasing of water demand
E.F-6: Competition between agricultural and non-agricultural sectors
E.F-7: Changing the quality of water resources due to discharge of wastewater (urban and industrial) into rivers and drains, especially in the central irrigation zone of the study area

* I.F: Internal factors related to agricultural water management.

**E.F: External factors related to agricultural water management.

### Determining the management alternatives for agricultural water demand and supply management

The management alternatives to improve the agricultural water demand and supply management in the irrigation management zones in the study area were determined to be: (1) Development/Rehabilitation of the *Sefidroud* irrigation network; (2) Improve the management of operation and maintenance of the *Sefidroud* irrigation network; (3) Wastewater management, and (4) Inter-basin water transfer within the *Sefidroud* irrigation network system (see Table 3).

**Table 3:** Agricultural water demand and supply management alternatives.

Executive policy		Management alternatives
Demand management	(1) Development/Rehabilitation/Renewing of the *Sefidroud* Irrigation Network	(1–1) Development and Implementation of the Main Irrigation and Drainage Network in the 7 Remaining Irrigation Units of the *Sefidroud* Irrigation Network
		(1–2) Rehabilitation/Renovation of the *Sefidroud* Irrigation Network in Under-Operation Irrigation Zones (10 Irrigation Units)
		(1–3) Development of On-Farm Irrigation and Drainage Network in the Remaining Areas of the *Sefidroud* Irrigation Network
		(1–4) Equipping and Renovating Paddy Lands in the Remaining Lands of *Sefidroud* Irrigation Network
		(1–5 Mechanization of Paddy Lands
	(2) Improve the Management of Operation and Maintenance of the *Sefidroud* Irrigation Network	(2–1) Strengthening the Irrigation Management Institutions of the *Sefidroud* Irrigation Network with Public Participation
		(2–2) Establishment of a Water User Association to Promote Stakeholders Participation in the form of Training, Holding Workshops and Upgrading the Capacities of Irrigation Management Institutions within the *Sefidroud* Irrigation Network
		(2–3) Supervision of *Sefidroud* Irrigation Network Operation by Consultant Engineers and Providing Documentation for the Irrigation Management Institutions
		(2–4) Establishment of Agricultural Water Use Monitoring System in the *Sefidroud* Irrigation Network
Supply management	(3) Wastewater Management	–
	(4) Inter-Basin Water Transfer within the *Sefidroud* Irrigation Network	(4–1) Development and Rehabilitation of small reservoirs in the *Sefidroud* Irrigation Network
		(4–2) Inter-Basin water Transfer within the *Sefidroud* Irrigation Network through the Construction of Diversion and Rubber Dams in the Downstream of Rivers and Drains

**Figure 5:**
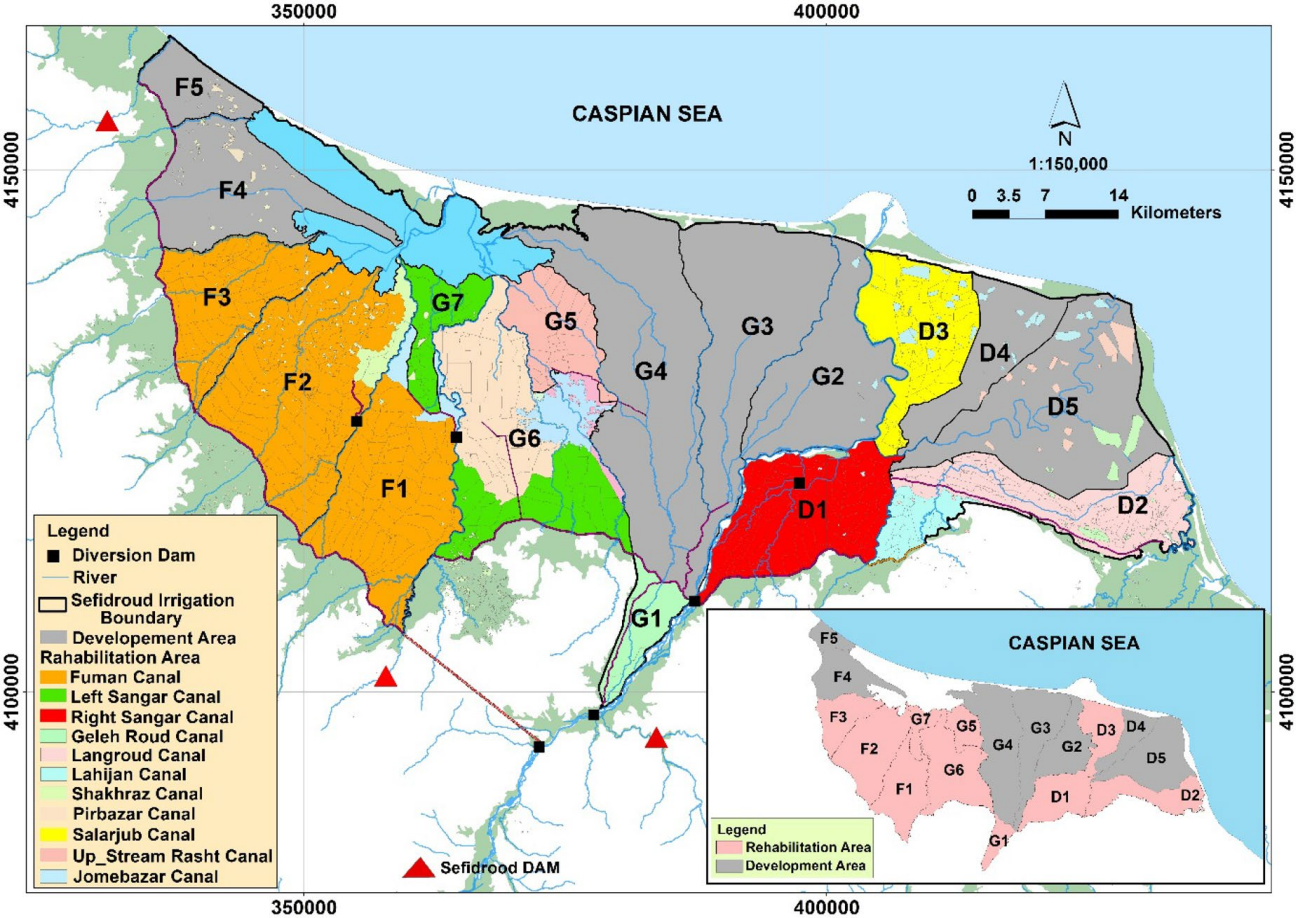
Spatial distribution of development and rehabilitation lands of the *Sefidroud* irrigation and drainage network. (Figure created in ArcGIS 10.4 ESRI, http://www.esri.com).

**Figure 6:**
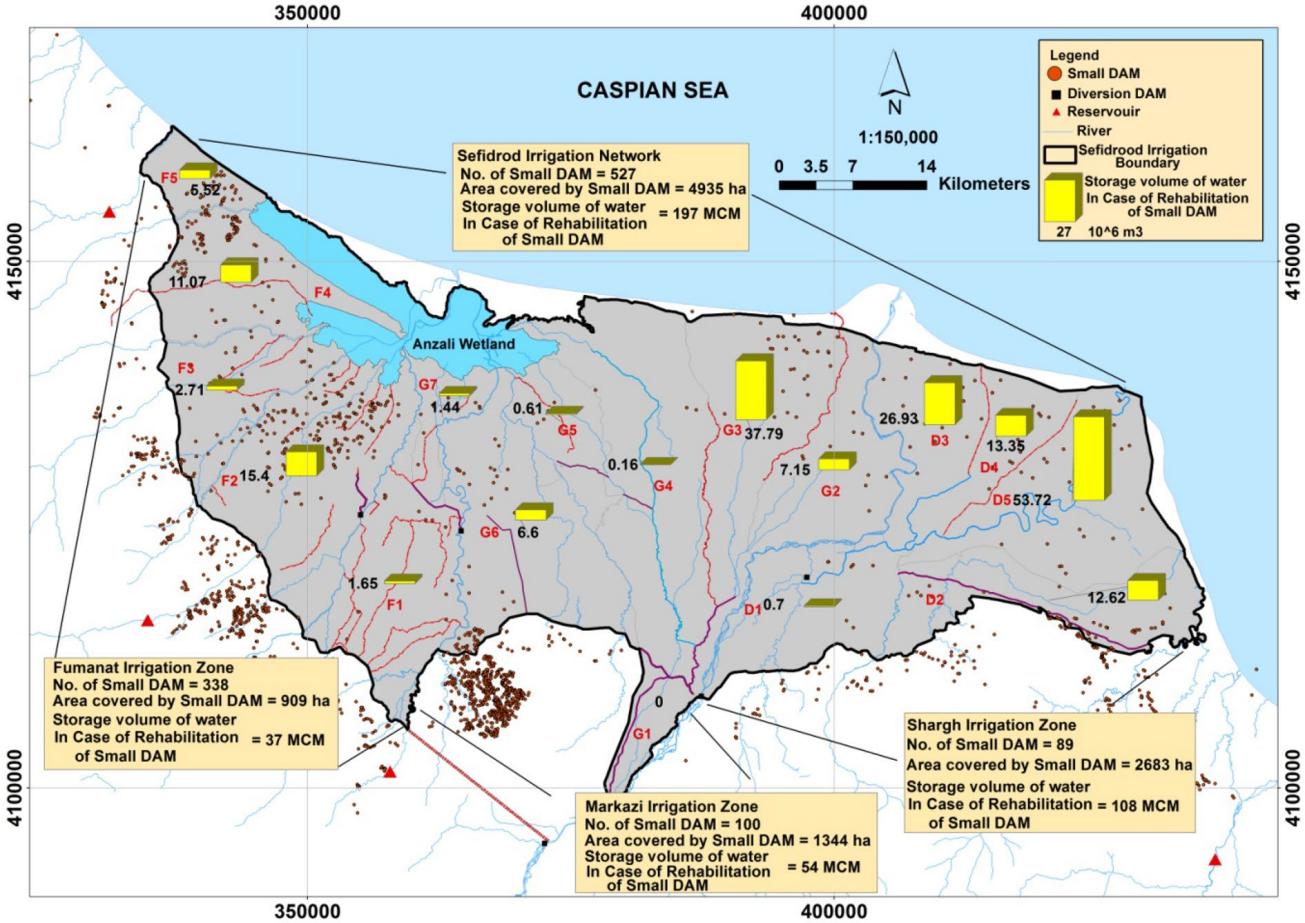
Spatial distribution of small irrigation reservoirs in the *Sefidroud* irrigation and drainage network corresponding to the rehabilitation and improvement conditions. (Figure created in ArcGIS 10.4 ESRI, http://www.esri.com).

**Figure 7:**
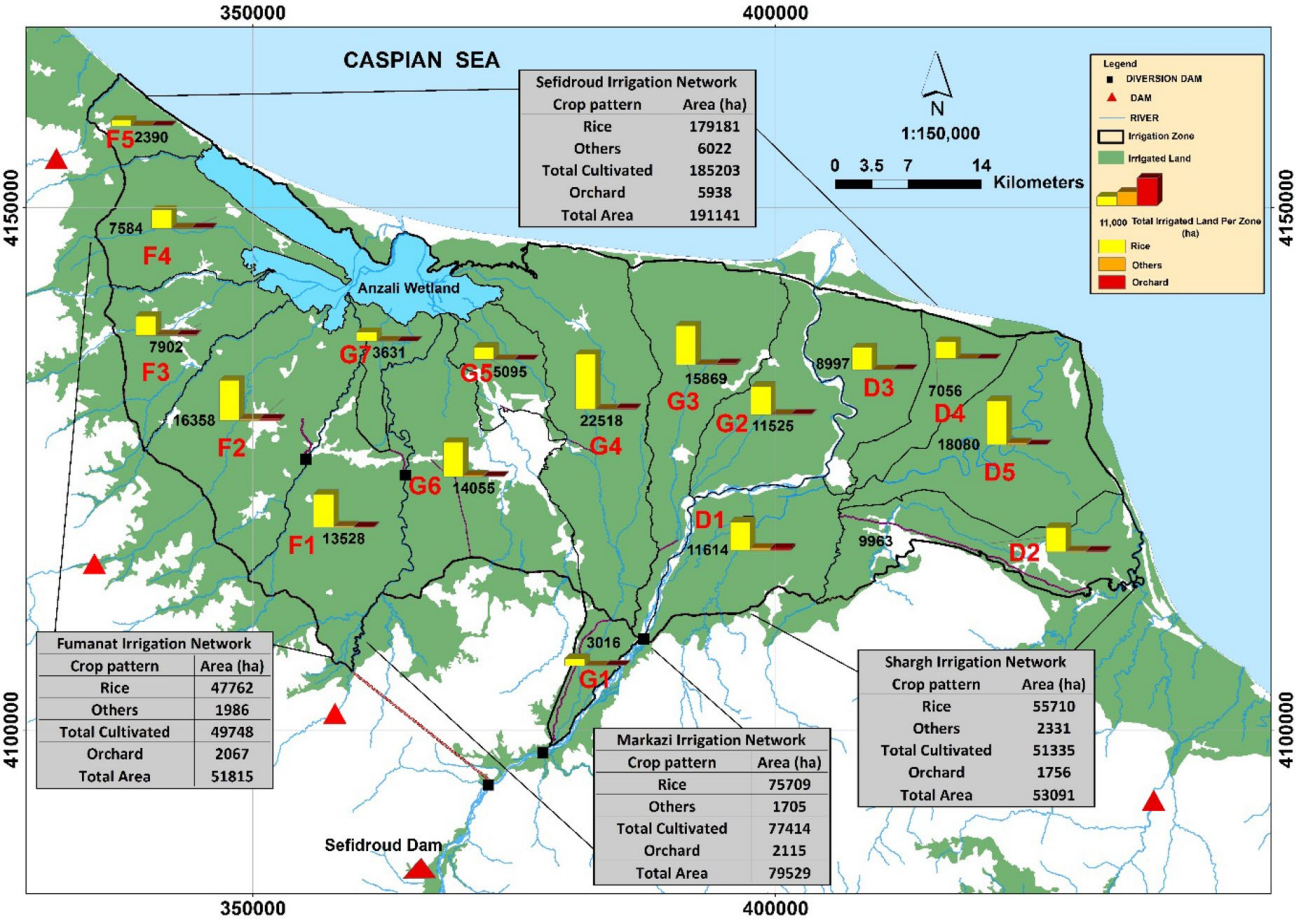
*Sefidroud* irrigation and drainage network land area corresponding to equipping and renovating conditions. (Figure created in ArcGIS 10.4 ESRI, http://www.esri.com).

**Figure 8:**
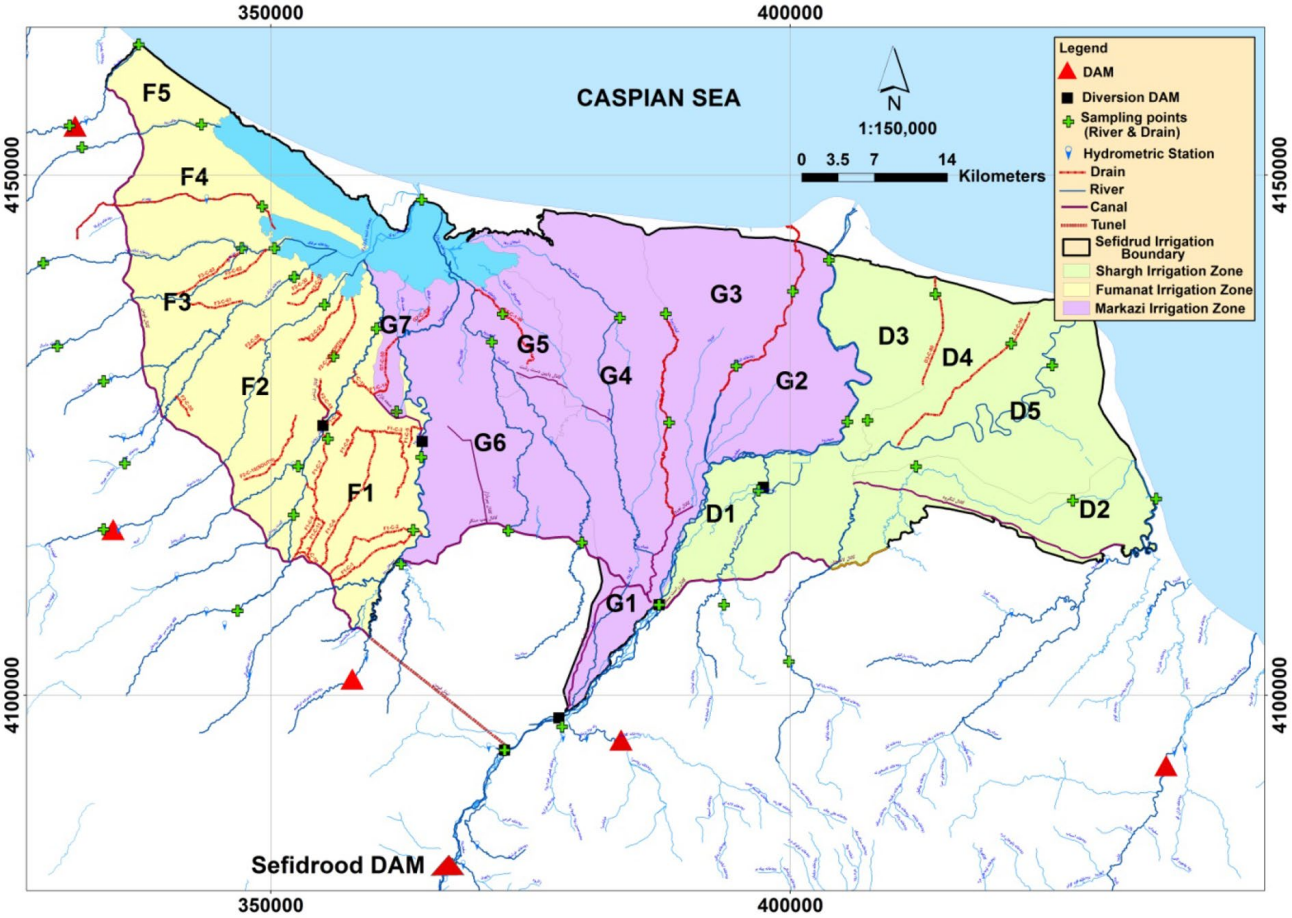
Location of the sampling points in local rivers and selected drainages. (Figure created in ArcGIS 10.4 ESRI, http://www.esri.com).

The spatial distribution of the management alternatives within the *Sefidroud* irrigation and drainage network were defined according to the management alternatives for agricultural water demand and supply management, and are shown in Figs. 5, 6, 7 and 8.

Under current conditions the management alternative of development/rehabilitation of the *Sefidroud* irrigation network’s infrastructure has not been fully implemented. Accordingly, completion and implementation of the main irrigation and drainage network in about 90,000 hectares represents one of the most important priorities in the *Sefidroud* irrigation network. Carrying out this management alternative would raise the irrigation efficiencies of the *Sefidroud* irrigation network. Furthermore, in spite of the implementation of the main irrigation and drainage network in 10 irrigation units of the *Sefidroud* irrigation network, the rehabilitation of the irrigation network in 102,000 hectares is imperative to achieve operational effectiveness. Figure 5 displays the spatial distribution of development and rehabilitation lands in *Sefidroud* irrigation network.

One of the effective management alternatives for maximum use of internal water resources in the study area is using the natural potential of small irrigation reservoirs existing in the *Sefidroud* irrigation and drainage network. The spatial distribution of small irrigation reservoirs is depicted in Fig. 6. It is seen in Fig. 6 that the total number of small irrigation reservoirs in the study area for agricultural water supply is equal to 527, and the total area of the small irrigation reservoirs is 4935 hectares. The total volume of stored water in small irrigation reservoirs is estimated at 197 million cubic meters under the rehabilitation and improvement conditions.

### Multi-criteria analysis of agricultural water demand and supply management

A fuzzy multi-criteria decision model was implemented to evaluate the agricultural water demand and supply management alternatives. The prioritization of the regional management alternatives in the *Sefidroud* irrigation and drainage network, which includes the *Markazi, Fumanat*, and *Shargh* irrigation zones, is accomplished with hierarchical analysis methods^56^ and the TOPSIS decision-making method in a fuzzy environment, which consists of the following stages^14^:

- Determining appropriate criteria for the decision-making process.
- Calculations related to the hierarchical analysis process.
- Evaluating the alternatives using the fuzzy TOPSIS model, and determining the final prioritization of alternatives.

The alternatives and criteria for decision making are determined and a hierarchical structure is formed. The hierarchical structure has a first level consisting of goals to be achieved, the second level consists of the decision criteria, and the third level consists of the management alternatives.

The weights of the criteria are determined by the hierarchical analysis method once the hierarchical structure is defined, which involves constructing a pairwise comparison matrix to determine the weights. The comparison matrix’s values are determined using Saaty’s table^55^, and the weights of the criteria are calculated based on the geometric mean values. The next step applies the fuzzy TOPSIS algorithm to evaluate the management alternatives in each of the irrigation management zones of the *Sefidroud* irrigation and drainage network. Lastly, the management alternatives are prioritized. The prioritization uses language variables to evaluate the management alternatives. The fuzzy TOPSIS calculates the CC_j_ indexes of the management alternatives, such that the alternatives’ rank or desirability increases with increasing value of the CC_j_ index. The CC_j_ index is a dimensionless metric in the range [0,1] that measures the closeness of a management alternative to an ideal management alternative or solution^29^.

#### Identifying the effective criteria in the decision-making process

The decision criteria are of central importance for evaluating the agricultural water demand and supply management alternatives. All the factors that are considered influential in the sustainable management of agricultural water must be studied. The decision criteria are studied separately for each of the irrigation management zones of the *Sefidroud* irrigation and drainage network to enable accurate decisions representing zonal conditions. Recall that three irrigation management zones of *Sefidroud* irrigation network are considered. The identified criteria are listed in Table 4.

**Table 4:** Criteria and indicators for evaluating agricultural water demand and supply management alternatives within the *Sefidroud* irrigation and drainage network.

Action plans	Criteria/index		Calculation	
(1) Development/rehabilitation/renewing of the *Sefidroud* irrigation network	Development of irrigation network	Development of main irrigation network	Area covered by main irrigation networks /total area of irrigation network	A.m/A.s
		Development of on-farm irrigation network	Area covered by on-farm irrigation networks /total area of irrigation network	A.o/A.s
		Equipment and renovating of irrigated lands	Area covered by equipment and renovating irrigation networks /total area of irrigation network	A.od/A.s
	Rehabilitation of irrigation network	Rehabilitation of irrigation units	Area of rehabilitated irrigation units/ total area of irrigation network	A.r/ A.s
(2) Improve the management of operation and maintenance of the *Sefidroud* irrigation network	Operation and maintenance		Annual cost for operation and maintenance of irrigation network / total cost for operation and maintenance of irrigation network	C.o&m/T.C.o&m
	Beneficiary participation	Development of water user association	No. of Beneficiaries under Water User Association/ No. Of Beneficiaries under Irrigation Network	N.b/N.T.b
	Water productivity	Water productivity (kg/m^3^)	Average amount of product per unit area/ average amount of water use per hectare	H/V
		Water productivity (Rial/m^3^)	Average product revenue per unit area / average amount of water use per hectare	I/V
(3) Inter-Basin water transfer within the *Sefidroud* irrigation network	Water saving	Regulating water from dams	Volume of agricultural regulated water within the study area/ The Total Volume of Agricultural Water Use in the Area	V.reg/T.Vc
		Regulating water from small irrigation reservoirs	Volume of Agricultural Regulated Water from Small Irrigation Reservoir / The Total Volume of Agricultural Water Use in the Area	V.T/T.Vc
(4) Wastewater management	Water quality	Quality of agricultural drains	The amount of pollutants in agricultural wastewater	Q.d

#### Evaluating the weights of the decision-making criteria and determining the final ranking of the management alternatives

**Figure 9:**
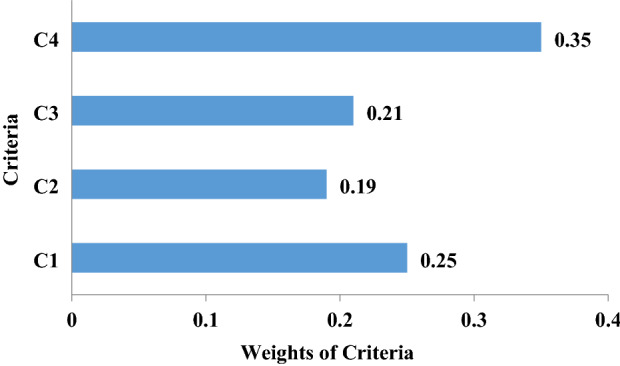
The results of the analytical hierarchical process (AHP). C1: social criteria, C2: economic criteria, C3: environmental criteria, C4: water use and resources management criteria.

The criteria listed in Table 4 were classified into four categories: C1 social criteria, C2 economic criteria, C3 environmental criteria, and C4 water consumption and resources management criteria. The calculated weights of the criteria are depicted in Fig. 9. The matrix of weighted fuzzy decision making was calculated using the weights of criteria obtained with the AHP method (Table 5). It is seen in Table 5 that the management alternatives A1 through A3 represent the management alternatives in the irrigation management zones, and C1 through C4 denote the decision-making criteria. Table 5 shows that the elements $\tilde{V}_{ij}$ for all values *i* and *j*, are normalized in the interval [0,1].

**Table 5:** The weighted fuzzy decision making matrix.

	C1	C2	C3	C4
A1	(0.100,0.150.0.100)	(0.038,0.076.0.114)	(0.280,0.350,0.350)	(0.042,0.084,0.126)
A2	(0.050,0.100,0.150)	(0.076,0.114,0.152)	(0.070,0.140,0.210)	(0.126,0.168,0.210)
A3	(0.200,0.250,0.250)	(0.114,0.152,0.190)	(0.140,0.210,0.280)	(0.000,0.042,0.084)
Ideal( +)	$\tilde{V}_{1}^{*} = \left( {1,1,1} \right)$	$\tilde{V}_{2}^{*} = \left( {1,1,1} \right)$	$\tilde{V}_{3}^{*} = \left( {1,1,1} \right)$	$\tilde{V}_{4}^{*} = \left( {1,1,1} \right)$
Ideal(-)	$\tilde{V}_{1}^{ - } = \left( {0,0,0} \right)$	$\tilde{V}_{2}^{ - } = \left( {0,0,0} \right)$	$\tilde{V}_{3}^{ - } = \left( {0,0,0} \right)$	$\tilde{V}_{4}^{ - } = \left( {0,0,0} \right)$

A1: *Markazi* irrigation zone, A2: *Shargh* irrigation zone, A3: *Fumanat* irrigation zone.

C1: social criteria, C2: economic criteria, C3: environmental criteria, C4: water use and resources management criteria.

The fuzzy positive ideal solution (FPIS, A*) and the fuzzy negative ideal solution (FNIS, A^-^) are defined as $\tilde{v}_{i}^{*} = \left( {1,1,1} \right)$ and $\tilde{V}_{i}^{ - } = \left( {0,0,0} \right)$, respectively, for use in the TOPSIS method with respect to the benefit criteria, The values of FPIS and FNIS are defined as $\tilde{v}_{i}^{*} = \left( {0,0,0} \right)$ and $\tilde{V}_{i}^{ - } = \left( {1,1,1} \right)$, respectively, for the cost criteria.

**Figure 10:**
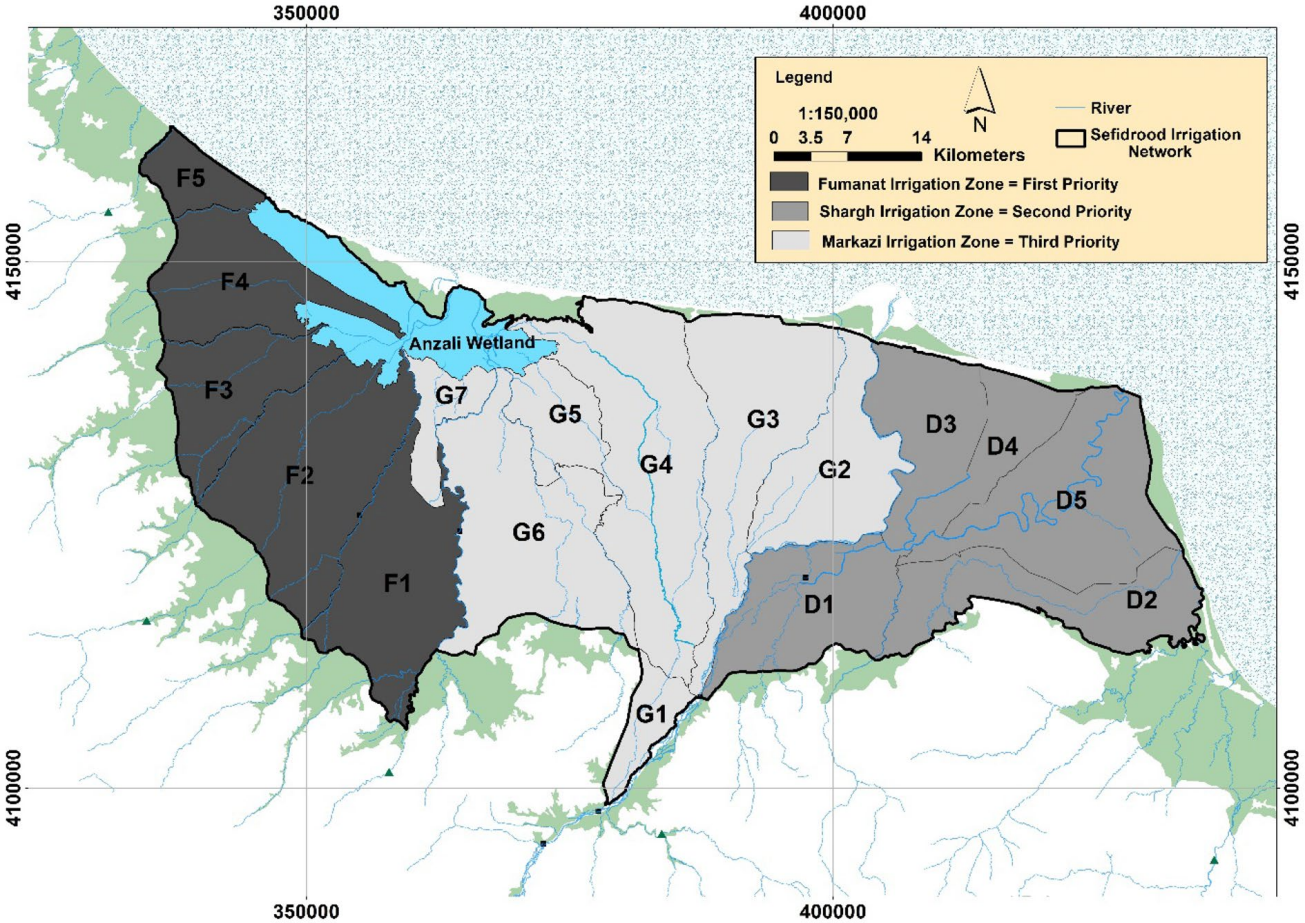
Prioritization of irrigation zones of the *Sefidroud* Irrigation and Drainage Network in terms of implementation of demand and supply management alternatives. (Figure created in ArcGIS 10.4 ESRI, http://www.esri.com).

All the criteria used in this work to rank of the agricultural water management alternatives are benefit criteria. The distance between the alternatives and the positive (D*) and negative (*D*^−^) ideals, and the CC_j_ indices are computed with TOPSIS^14^. The calculation results for the CC_j_ index are listed in Table 6, where it is seen that alternative A3 (*Fumanat* irrigation zone) with CC_j_ index equivalent to 0.151 is selected as a first priority (top rank) as an agricultural water-demand and supply-management regional management alternative. The lowest priority (bottom rank) of alternatives based on the CC_j_ index is listed in Table 6. The prioritizing of other alternatives based on CC_j_ values is also shown in Fig. 10.

**Table 6:** The ranking of alternatives based on the CCj index.

Alternatives	*D*_i_*	*D*_*i*_^−^	*CC*_*i*_	_Final ranking of alternatives_
A_1_	3.442	0.586	0.145	3
A2	3.426	0.603	0.150	2
A3	3.422	0.611	0.151	1

A1: *Markazi* irrigation zone, A2: *Shargh* irrigation zone, A3: *Fumanat* irrigation zone.

#### Sensitivity analysis

**Figure 11:**
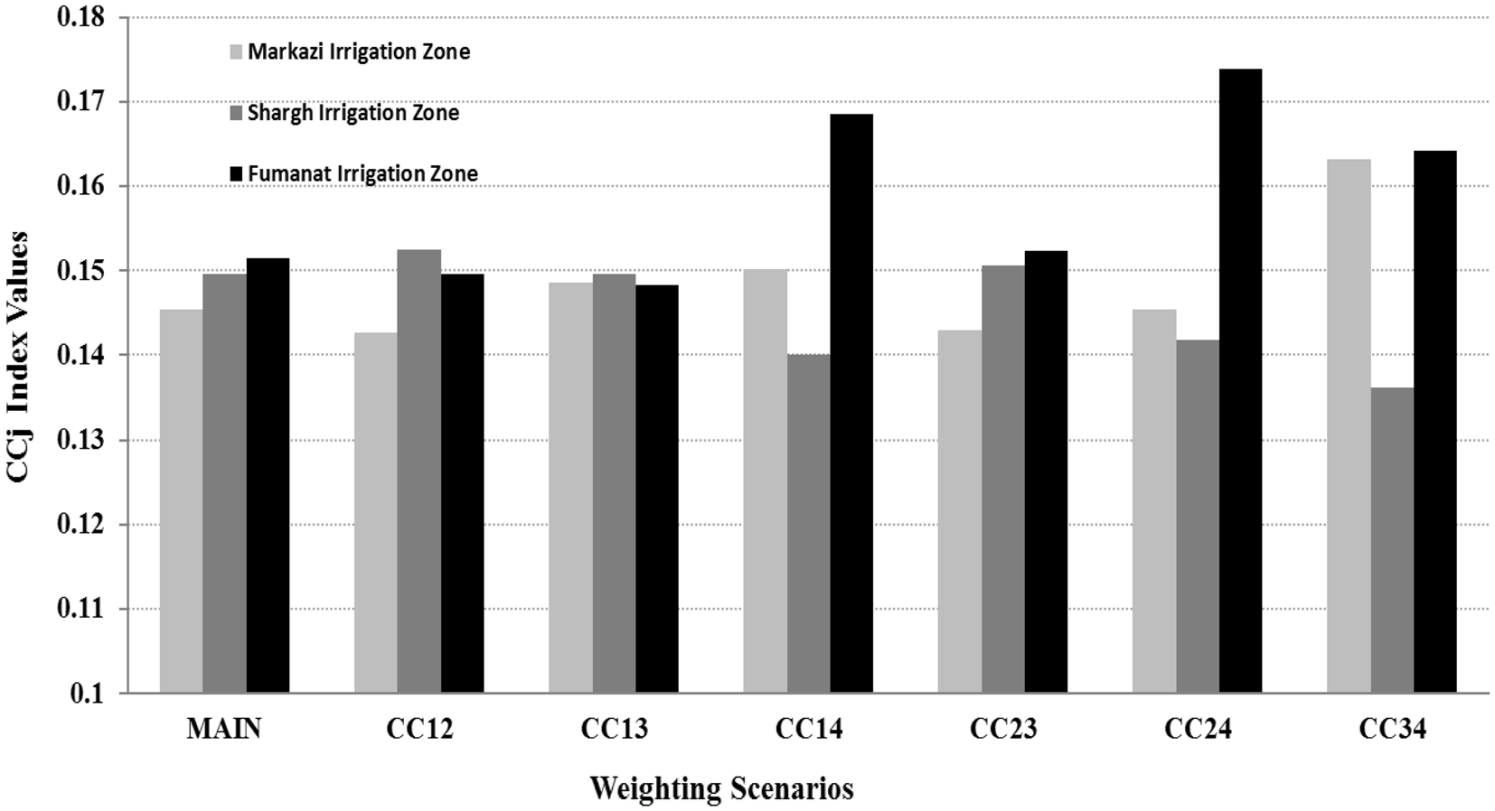
Sensitivity analysis of the model with respect to the weighting scenarios.

The end product of the multi-criteria analysis process consists of proposing an alternative or a set of alternatives for implementation. Sensitive numerical inputs that may have a major impact on the final decision (i.e., the ranking of alternatives) must be identified. The purpose of sensitivity analysis is to determine how the proposed alternatives are affected by changes in inputs (i.e., criteria weights). This analysis evaluates the robustness, or lack of it, of the proposed solution. The sensitivity analysis is performed by changing the weights of the decision making criteria. There are seven combinations of the weights, each defining a weighting scenario, which are listed in Table 7. The results of the sensitivity analysis of the model are listed in Table 7 and Fig. 11. The values of the criteria weights, which are determined from multi-criteria analysis, are listed in Table 7 and Fig. 11. The CC_j_ values corresponding to the six weighing scenarios are listed in Table 7. CC_23_, for example, represents a scenario in which the weights of the second and third criteria are changed. It can be seen in Table 7 that the sixth scenario, in which the weights of the second and fourth criteria are changed, establishes that alternative A3 (*Fumanat* irrigation zone) has the largest CC_j_ value equal to 0.174 compared to its initial value of 0.151. The scenario, in which the weight of the third and fourth criteria are changed, establishes that alternative A1 (*Markazi* irrigation zone) has the larger CC_j_ value equal to 0.163 compared to its initial value of 0.145. Also, the second scenario, in which the weight of the first and second criteria are changed, indicates that alternative A2 (*Shargh* irrigation zone) has the largest CC_j_ value equal to 0.152 compared to the initial value of 0.50.

**Table 7:** Sensitivity analysis of the results.

Scenario	Weights of criteria				CCj index values per alternatives		
	W_1_	W_2_	W_3_	W_4_	A1	A2	A3
1 = Main	0.25	0.19	0.21	0.35	0.145	0.150	0.151
2 = CC_12_	0.19	0.25	0.21	0.35	0.143	0.152	0.150
3 = CC_13_	0.21	0.19	0.25	0.35	0.149	0.150	0.148
4 = CC_14_	0.35	0.19	0.21	0.25	0.150	0.140	0.169
5 = CC_23_	0.25	0.21	0.19	0.35	0.143	0.151	0.152
6 = CC_24_	0.25	0.35	0.21	0.19	0.145	0.142	0.174
7 = CC_34_	0.25	0.19	0.35	0.21	0.163	0.136	0.164

A1: *Markazi* irrigation zone, A2: *Shargh* irrigation zone, A3: *Fumanat* irrigation zone.

W1: Weight of social criteria.

W2: Weight of economic criteria.

W3: Weight of environmental criteria.

W4: Weight of water use and resources management criteria.

## Concluding remarks

This work develops and applies a conceptual framework of strategic planning and multi-criteria decision making for sustainable agricultural water management. Also an analytical model for estimating agricultural water use based on multiple factors was developed. The results of the agricultural water use analysis and the identification of internal and external factors affecting the management of agricultural water resources led to defining regional management alternatives for agricultural water demand and supply.

Decision making involves the use of a method that accounts for uncertainty within the decision-making process. Hence, a model of decision making with regard to the alternatives (the irrigation management zones of the *Sefidroud* irrigation and drainage network) was developed based on four water resources sustainability criteria: social, economic, environmental, and water use resource management. The presented framework combines the method of analytical hierarchy and the fuzzy TOPSIS, which permits taking into account the effect of the criteria weights in multi-criteria decision making. The study’s results showed that alternative A3 (the *Fumanat* irrigation zone) was top ranked (first priority) among other irrigation zones as the best regional management alternative. The sensitivity analysis results have demonstrated that in five among seven scenarios the *Fumanat* irrigation zone was ranked first with respect to regional management alternatives for agricultural water demand and supply. The framework developed in this work can be applied to other large scale water resources system in which regional differentiation is essential for sustainable water management.

## Data Availability

All relevant data are included in the paper or its supplementary information.
